# Perspectives of educators and students on the efficacy of online teaching and learning strategies employed during COVID-19 in a health sciences institution

**DOI:** 10.1007/s11845-024-03773-8

**Published:** 2024-08-16

**Authors:** Keith Geraghty, Nicole Heng, Juliette Duff, Jacinta Burke, A. D. K. Hill, John Jenkins, Gozie Offiah

**Affiliations:** 1 Department of Surgery, Royal College of Surgeons in Ireland (RCSI), University of Medicine and Health Sciences, Dublin, Ireland; 2 Medical Student, Royal College of Surgeons in Ireland (RCSI), University of Medicine and Health Sciences, Dublin, Ireland; 3 Department of CoMPPAS, Royal College of Surgeons in Ireland (RCSI), University of Medicine and Health Sciences, Dublin, Ireland; 4 Director of THEP, Royal College of Surgeons in Ireland (RCSI), University of Medicine and Health Sciences, Dublin, Ireland

**Keywords:** Pandemic, Students, Teaching and learning strategies, University

## Abstract

**Background:**

Healthcare education encountered unprecedented challenges during the COVID-19 pandemic, but the necessary responses have also provided learning opportunities for the future.

**Aims:**

We aimed to evaluate the impact of COVID-19 on teaching and learning strategies and the perceptions of clinical educators and health sciences students on novel methods to improve online student engagement in Ireland’s largest medical school.

**Methods:**

Two separate online surveys designed to gain insights into the perceived efficacy of online teaching strategies were distributed to clinical educators and health sciences students (medical and pharmacy) over 7 months via email.

**Results:**

A total of 86.4% of educators responded that rapport was more difficult to build online, and 90.5% reported less engagement from students online. The most popular methods that improved student engagement included using polls, a chat box function for questions, small group discussions and having student cameras turned on. Amongst educators surveyed, 81.8% felt a training course focused on teaching strategies at the start of every academic year would be beneficial. From the students’ perspective, no difference was noted between the medicine and the pharmacy students. Seventy-five percent reported using quizzes/polls, and 63% reported using game-based platforms as techniques to improve online learning. Sixty-two percent of students described it as a positive outcome of learning during the pandemic.

**Conclusion:**

Any pandemic poses unique challenges to the delivery of healthcare education. These surveys report educators’ and students’ views on online teaching and learning strategies, highlighting novel mechanisms to improve student engagement and ultimately impact on graduate outcomes.

**Supplementary Information:**

The online version contains supplementary material available at 10.1007/s11845-024-03773-8.

## Introduction

The COVID-19 pandemic has caused unprecedented challenges to healthcare provision and health professions education. Healthcare professional students were affected when traditional pedagogical methods of clinical placements and large-group lectures were cancelled or restricted to halt the spread of COVID-19 [1]. Universities had to adapt to this new environment, ensuring the highest standard of education for students while simultaneously assuring safety [2]. This required curricular and examination adaptations to facilitate social distancing measures. Before the pandemic, virtually all health professions education in the Royal College of Surgeons in Ireland University of Medicine and Health Sciences (RCSI) was delivered in person, except for students on remote attachments who tuned in online for large group teaching sessions in the main teaching hospital. Courses were delivered via face-to-face tutorials and lectures, with infrequent use of the online medium. When the lockdown occurred, there was a stark change in the delivery of education in RCSI. Using online teaching and technology was paramount to facilitating health professions education during the COVID-19 pandemic [3]. The sudden, unexpected requirement for a remote health professions curriculum posed many challenges and opportunities to schools and their students with a need for urgent adaptation [4–10].

### The impact of COVID-19 pandemic on health profession education

While it was unprecedented and led to massive disruption to traditional teaching methods, COVID-19 pandemic brought virtual web-based learning to the forefront of health profession education as programmes adapted to physical distancing challenges while maintaining the rigorous standards of training. The COVID-19 pandemic introduced innovative ways of teaching and learning under duress with traditional teaching replaced with e-learning to enable continuity of learning. E-learning introduced positive perceptions focused on technology access, possession of basic computer skills, pedagogical design of online courses, online interactions and learning flexibility as described in a systematic review of 250 studies with a total of 111,622 students [11]. Frenk et al. in the lancet also described the transformative development in health-professional education that were expedited as a result of the COVID-19 pandemic, for example, the issues of globalisation of health care, increasing concerns of health disparities across the world and the use of large-scale application of information technology to education [7]. A recent commentary in Academic medicine by Sklar emphasised how the pandemic created a need to re-examine the current curricular emphasis on the bio scientific model of health and to broaden the educational approach to incorporate the behavioural, social and environmental factors that influence health [1]. Other studies explored the impact of online education on students and teachers in health professional courses including Medicine, Nursing, Allied Health and Biomedical Science, the longitudinal outcome of student learning attributes and impact on graduate outcomes and thus the perceived efficacy of online teaching and learning strategies employed during pandemic for health professions’ education [8]. These studies emphasise the need for adaptability on the part of educators and learners with online learning [12].

### Clinical educators in a health science institution

RCSI has a unique cohort of interdisciplinary clinical educators. Clinical educators ranged in grade from post-internship to consultant in over 12 clinical departments, some of which are Anaesthetics and Critical Care, Clinical Microbiology, Trauma and Orthopaedics, Surgery, Medicine, Psychiatry, Paediatrics, Obstetrics and Gynaecology and other disciplines like Pharmacy. Our students learn in a truly immersive environment where clinical educators facilitate student learning in the workplace environment [13]. The clinical educator role has been recognisable for decades, with a face-to-face, hands-on approach to achieving student competence and skill acquisition. Educators utilised student-centred learning, team-based, cooperative and interdisciplinary methods to integrate the students into their future work environments [13]. Clinical educators are aware of the importance of their role and aspire to achieve the most optimal training for the student cohort. The importance of this relationship is confirmed in the literature, where the relationship between trainee and educator has been described as the most important factor for supervisory effectiveness [14]. The experience of being a clinical educator with a sense of self-identity, a sense of relationship with others, a sense of purposeful action and a sense of being a clinical educator has been well described in the literature [15]. Due to the lockdown effect on teaching, the educator-student relationship and the identity of being a clinical educator were greatly impacted. Clinical educators had to adapt to novel methods of teaching clinical as well as complex communication, technical and empathy skills to future healthcare professionals.

### Diverse study body in a health science institution

In addition to a diverse clinical educator cohort, RCSI has one of the most diverse multicultural student populations, with an international community of students from over 95 countries. This diverse student population means various skills, and learning needs that must be catered to ensure RCSI graduates can serve diverse people and cultures and deliver healthcare in a complex global environment.

Through a survey of clinical educators and students at RCSI, our study aimed to explore the key learning points from the transition to online education during COVID-19 in an Irish context. During the pandemic, RCSI adopted several online teaching methods, which have been retained [16]. As such, this study aimed to determine the perceived challenges COVID-19 imposed on health professions education from both the clinical educators’ and students’ perspectives while identifying e-learning strategies in response to these issues. These techniques are pivotal for the progress of online teaching methods and the future of an evolving health professions education workforce and landscape.

## Methods

### Setting

This research study was conducted in the context of undergraduate medical and pharmacy education at RCSI, University of Medicine and Health Sciences. RCSI is a dedicated Health sciences university with a School of Pharmacy, Physiotherapy, and Medicine. RCSI has the largest medical school in Ireland, with approximately 1800 students across the 5-year direct entry programme and graduate entry programme. RCSI also has campuses in the mid-east and Asia, focusing on transnational curriculum delivery.

### Study design

Two separate electronic cross-sectional surveys were distributed to the clinical educator cohort of 120 people across the 12 clinical departments in the School of Medicine and to School of Pharmacy and the student population of RCSI to include Medical and Pharmacy students. Research ethics committee approval was attained before survey distribution. Informed consent was obtained from all participants and recorded electronically during the survey process. Survey data were processed anonymously, confidentially and in compliance with the General Data Protection Regulations of the European Union (GDPR). The electronic cross-sectional surveys were sent by a gatekeeper, the Quality Enhancement Office (QEO) in RCSI. The QEO emailed staff, and data was gathered via Survey Monkey (San Mateo, CA, USA), an online survey distribution and management platform. The survey started on 18 May 2021; several reminders were sent to both cohorts from the Quality Enhancement Office in an effort to generate a larger sample size and to increase response rate. The survey data collection ended on 10 December 2021. Surveys were emailed by QEO at defined periods between May and December, so it did not clash with the end of rotation or end of year surveys. The surveys are available in supplementary materials.

The surveys were conducted in English and took approximately 25 min to complete.

A survey-based methodology was chosen due to administrative convenience. The surveys were designed using dichotomous, multiple choice, Likert response scale and open-ended questions. The survey was created by students and educators from course evaluation and informal feedback. The survey questions were reviewed and piloted by a group of educators and students who were randomly selected by the research group. The survey were piloted ahead of wider dissemination to test the research process and ensure the survey items adequately addressed the research question. The student survey comprised 36 items, and the clinical educator had 48 items.

Data collected on Survey Monkey was transferred to Excel (Microsoft, Redmond, Washington, USA) and analysed in StataCorp, 2021, Stata Statistical Software: Release 17. Initially, descriptive statistics were used to describe the characteristics of the sample. Continuous variables were summarised using means and standard deviation, and categorical variables were summarised as frequencies and percentages. The authors reviewed open-ended responses, and themes were identified.

## Results

### Student survey

A total of 3.35% (*n* = 67 out of 2029)^1^ of students across direct entry and graduate entry medicine and pharmacy responded to the survey. Of participants, 28.4% (*n* = 19) were male and 71.6% (*n* = 48) were female. Of students, 44.8% (*n* = 30) surveyed were in pre-clinical years, and the remainder (55.2%, *n* = 37) were in clinical years. Table 1 shows the demographics of the students surveyed.

**Table 1:** Student demographics

	% (*n*)
Gender	
Male	28.4 (19)
Female	71.6 (48)
Age range	
18–24 years	67.2 (45)
25–34 years	31.3 (21)
45–54 years	1.5 (1)
Course of study	
Medicine	85.1 (57)
Pharmacy	14.9 (10)
Student ethnicity	
White and White Irish	50.7 (34)
Arab	19.4 (13)
Indian/Pakistani/Bangladeshi	10.4 (7)
Asian	9.0 (6)
Chinese, Malay and Malay Chinese	7.5 (5)
Black African/Black Irish	3.0 (2)

Of the students that responded to the survey, 51.4% (*n* = 35) of both medicine and pharmacy students reported that RCSI supported their needs concerning the COVID-19 pandemic, whereas 13.2% of respondents (*n* = 9) felt their needs were unmet. One hundred percent (*n* = 67) of students reported having their PC/laptop/device for study or online learning. Of students, 67.7% (*n* = 46) stated they found it easy to access online classes and learning materials, whereas 8.8% (*n* = 6) of students did not find the material easy to access.

When questioned on ‘I felt motivated when working from home’, 25.4% (*n* = 17) of students strongly agreed or agreed, while 34.3% (*n* = 23) disagreed or strongly disagreed. When asked if they ‘felt comfortable engaging during online classes’, 44.8% (*n* = 30) strongly agreed or agreed, while 17.9% (*n* = 12) disagreed or strongly disagreed. When asked about the ideal time for an online session, 50.7% (*n* = 34) of both student cohorts responded 45 min, and 23.9% (*n* = 16) replied 1 h.

Techniques students perceived as improved online learning can be seen in Table 2. All students answered this question, with 41.8% selecting four or five techniques out of the seven options. Of all the students (100% of pharmacy students), 77.6% chose the use of polls/ungraded quizzes during lectures as the best technique to improve students’ experience and engagement, 62% chose game-based platforms to test students’ knowledge during teaching, and 55.2% chose the use of actors or other educators such as a simulated patient for students to practice history taking or other communication skills.

**Table 2:** Student responses when asked to select techniques that improve a student’s online learning experience

Technique	Students (*n*)	Percentage (%)
Breakout rooms	19	28.4
Polls/ungraded quizzes	52	77.6
Cluster lectures to earlier in the day	29	43.3
Flipped classroom^a^	19	28.4
Distribute case before the tutorial	27	40.3
Use of actors for simulated history-taking	37	55.2
Game-based platforms to test knowledge	42	62.7

^a^Flipped classroom is an approach to teaching and learning that inverts the structure of the traditional classroom. It is a type of blended learning where students encounter information before class, freeing class time for activities that involve higher order thinking in an interactive and experiential way

**Fig. 1:**
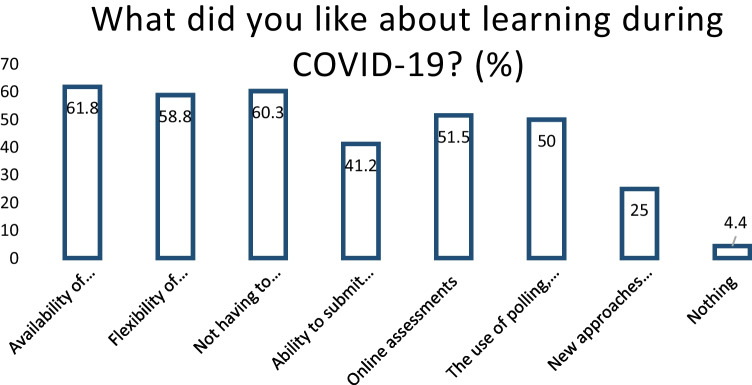
Student responses to ‘What, if anything, did you like about learning during COVID-19?’

When asked to choose the top 3 (out of seven options) challenges to learning during the COVID-19 pandemic, 79.1% of the students who completed the survey answered this question. 52.2% (*n* = 35) of students reported feeling ‘isolated and disconnected from peers’, 52.2% (*n* = 35) of students voted ‘limited availability of clinical experience’ and 49.3% (*n* = 33) of students selected ‘difficulty when staying motivated to study at home’ (Table 3). Student opinions on factors that were positive outcomes of learning during COVID-19 are represented in Fig. 1 below.

**Table 3:** Student responses when asked to choose ‘Top 3 challenges to learning during COVID-19’

Challenges to learning	Students (*n*)	Percentage (%)
Difficulty staying motivated to study at home	33	49.3
Feeling isolated and disconnected from peers	35	52.2
Less opportunities to contact teaching staff	12	17.9
Online lectures are less engaging than in-person	19	28.4
Lack of suitable study space at home	12	17.9
Limited availability of clinical experience	35	52.2
Mental health difficulties impact learning	26	38.8

**Fig. 2:**
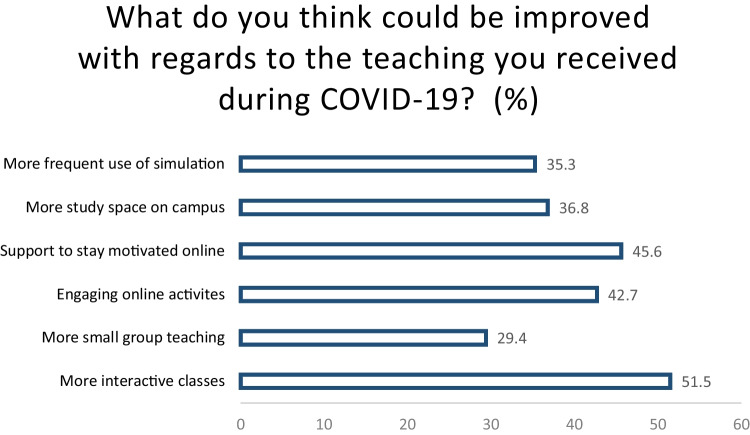
Student responses to ‘What do you think could be improved regarding the teaching you received during COVID-19?’

In a separate, open-ended question, 80.6% of the survey students completed the question. Of the 80.6%, 25% (*n* = 17) of students mentioned ‘online/blended learning’ or ‘recorded lectures’ as a suggested improvement for future curricular design after their experience during the COVID-19 pandemic, as noted in Fig. 2 below.

### Clinical educator survey

Of clinical educators, 23.3% (*n* = 28) responded to the survey. Of respondents, 60.7% (*n* = 17) were female, while 35.7% (*n* = 10) were male, and 3.6% (*n* = 1) preferred not to disclose gender. Participants reported teaching a range from first-year students to internship level. Of tutors, 78.6% (*n* = 22) taught online and in-person during COVID-19.

Educators reported that using student names and non-syllabus-related conversations before teaching improved camaraderie. A total of 67.9% (*n* = 19) reported reduced student engagement when teaching online. Of educators, 35.7% (*n* = 10) described using breakout rooms when teaching online, with 70% (*n* = 7) of those using them finding breakout rooms improved levels of student interaction. Forty percent (*n* = 4) of tutors found that breakout rooms helped identify parts of the syllabus that students found difficult. 10.7% (*n* = 3) of clinical educators utilised ungraded quizzes during their teaching, with 100% of those ‘strongly agreeing’ that their use improved student interaction. A total of 28.6% (*n* = 8) responded that they used the flipped classroom method, with 75% of those ‘agreeing’ it improved student engagement (Table 4). Fifty percent (*n* = 14) of instructors provided recorded lectures for students. In the open-ended question about the advantages and disadvantages of recorded lectures, the responses had similar themes; the advantages were ‘flexibility for student learning’, and the disadvantages mainly focused on ‘poor engagement’ and ‘poor attendance’ from students during live lectures when lecture recordings were made available. Of respondents, 86.4% (*n* = 19) stated that building rapport when teaching online was harder. Fifty percent (*n* = 11) of replies noted a change to their teaching style in response to the pandemic. Of educators, 81.8% (*n* = 18) felt a ‘train the trainer course focusing on teaching strategies would be beneficial at the start of the year’.

**Table 4:** Clinical educators reported utilising techniques for online teaching and perceived effects on student interaction

Technique	Educators % (*n*)	Positive effect (%)	Negative effect (%)	Neutral (%)
Breakout rooms	35.7 (10)	70	10	20
Ungraded online quizzes	10.7 (3)	100	0	0
Flipped classroom	28.6 (8)	75	0	25
Recorded lectures*	50 (14)	50	50	0

Positive effect = strongly agree/agree improved student interaction on the Likert response scale. Negative effect = strongly disagree/disagree improved student interaction on the Likert response scale. Neutral = neutral on the Likert response scale.

*Recorded lectures positive/negative effect = discerned from open-ended responses regarding advantages/disadvantages of recorded lectures.

## Discussion

Our study explored the perception of clinical educators and students on the efficacy of online learning. The two surveys in our study report views from two unique groups involved in health science education in Ireland: a diverse clinical educator cohort and a multicultural medical student population. The findings in both surveys portray COVID-19 as having a negative impact on health professions education. 86.4% of educators stated that building rapport with students was more difficult, while students’ main concerns were restricted clinical experience (51.5%) and feelings of isolation when learning from home (51.5%). One corresponding difficulty was highlighted in both surveys — engagement when learning online. This issue has been extensively reported in the literature [2, 17, 18]. Interestingly, there was one technique that both groups reported improved student engagement when teaching online — the use of ungraded polls and quizzes. The pandemic increased the reliance on recording of lectures. Over half of the students claimed recorded lecture material was beneficial to learning while the educators were divided on this matter. Contemporary techniques in education, such as the flipped classroom and breakout rooms, were seen positively by 75% and 70% of tutors, respectively, whereas we found that only 27.9% of students reported positive views on both methods.

Henderson et al*.* described student-reported benefits of online learning. The authors highlighted student benefits from online learning as flexibility of location (32.7%), time-saving (30.6%) and reviewing, replaying and revising material (27.9%) [19]. This is in keeping with our findings, with 58.8% of students saying the ‘flexibility of online/blended learning’ was advantageous. The flipped classroom has been shown to benefit student performance in medical examinations [13] and has reports of high levels of satisfaction from medical students [14]. In medical education literature, the use of polls is mentioned sparsely. Hilburg et al*.* describe the usefulness of the poll function as an ‘ice-breaker’ or to test knowledge [17]. In broader online educational research, students have reported quizzes as the most interactive online tool [20]. Breakout rooms were identified as a beneficial tool for teaching medical students in a 2020 study by Rucker et al. [21].

Several studies have shown that COVID-19 has had a negative effect on health professions education from both the student and educator perspectives [2, 17–22]. Students highlighted feelings of isolation and disconnection from their peers, struggled with engagement with study and felt they lacked clinical experience. The negative effects reported by clinical educators were a lack of student engagement, difficulty building rapport and poor student attendance online.

However, while the results of this study are in keeping with these, there were numerous outcomes linked to the transition to online learning, which we identified as positive. For students, the main benefits reported were the flexibility of blended learning, not having to travel to campus and the availability of recorded lecture material. Clinical educators adapted to the new teaching environment, utilising online teaching techniques (polls/quizzes, flipped classrooms and breakout rooms) to improve student engagement with good effect.

The limitations of this study are mainly related to the sample size and the use of a non-validated survey, which is beyond the scope of this study as the primary aim of the study was to gain insight into perceived challenges faced from moving online due to the pandemic. Although both surveys were distributed to a large sample population, the response rates were relatively low (3.35% for students and 23.3% for clinical educators).

## Conclusion

This study highlights strategies to improve student involvement when delivering online education. Polls and quizzes were unanimously viewed as beneficial amongst educators and students. Other techniques, such as breakout rooms and the flipped classroom, were favoured by tutors. The use of recorded material and online/blended learning was viewed as advantageous by students; however, it had mixed reviews from the trainer cohort. These findings can be applied to improved student involvement during crisis times such as the COVID-19 pandemic and in the progression of health professions education towards increased online learning. Online learning has been a pivotal tool for providing health professions education and this study has highlighted the student and educator views on the methods to successfully improve online learning. The main finding we can report is the unanimously positive view towards ungraded polls and quizzes to enhance student engagement during online teaching. Further prospective studies to assess online teaching methods are warranted, as the future of health professions education will likely follow the path of blended learning in an ever-increasingly digital world. Finally, this study highlights the need for student engagement during curriculum design and implementation considering the variation in opinion between students and educators.

## Supplementary Information

Below is the link to the electronic supplementary material.

Supplementary file1 (DOCX 36.5 KB)

Supplementary file2 (DOCX 38.0 KB)

## Data Availability

The raw data supporting the conclusions of this article cannot be made available by the authors because they do not have ethical approval to share these data. Further inquiries can be directed to the corresponding author.

## Footnotes

1 Of the 2029 students, 1800 are medical students.
