# Supplementary material for: Perspectives of educators and students on the efficacy of online teaching and learning strategies employed during COVID-19 in a health sciences institution
Source: Ir J Med Sci. 2024 Aug 16;193(6):3045–51. doi: 10.1007/s11845-024-03773-8 (PMC11666614; doi:10.1007/s11845-024-03773-8)
Supplement: Supplementary file 1 — Supplementary file1 (DOCX 36.5 KB) [file 11845_2024_3773_MOESM1_ESM.docx]

Many thanks for agreeing to participate in the study entitled **“The impact of COVID-19 on the teaching and learning strategies in a Health Sciences institution”.**

RCSI students, like all university students around the world, have adapted to a new way of learning throughout the COVID-19 pandemic. After a year of remote learning and significantly reduced in-person teaching, we want to hear from RCSI students about their experiences, what worked well or did not work well. As we learn to live with Covid-19 we are now looking to the future and how we can create valuable and positive learning experiences during a very challenging time. We hope that the data gathered will help us to better understand students’ needs and the challenges they face when learning during a pandemic. Thus guiding RCSI as it aims to provide a superb university education and equip students with all the knowledge and skills they need as they enter their chosen field.

**Principal investigator**: Gozie Offiah, Senior Lecturer, Department of Surgery, RCSI

**Co-investigator**: Juliette Duff, Tutor, Department of Surgery, RCSI

You will find a copy of the Participant Information Leaflet attached to the email accompanying this questionnaire.

The survey itself will follow after the consent form, and should take 10-15 minutes to complete.

**Consent Form**

Having read the information leaflet sent to you by email, please confirm your consent by selecting Yes or No for each the following questions:

- I have read and understood the Information provided about this research project.

Yes No

- I understand that my participation is voluntary.

Yes No

- I understand that I don’t have to take part in this study and that I can opt out at any time. I understand that I don’t have to give a reason for opting out and I understand that opting out won’t affect my education in RCSI.

Yes No

- I understand that I will not be paid for taking part in this study.

Yes No

- I am aware of the potential risks and benefits of this research study.

Yes No

- I consent to take part in this research study having been fully informed of the risks and benefits.

Yes No

- I give informed consent to have my data processed as part of this research study.

Yes No

- With respect to possible future research related to the current study, please answer A or B here below:

1. I do not consent for this data to be used for other research studies in the future
2. I give permission for data to be stored for possible future research without further consent being required but only if the research is approved by a Research Ethics Committee

**Demographics:**

**1**. What is your course of study? (e.g. Pharmacy, Undergraduate Medicine etc.)

**2.** What year of study are you?

- 1
- 2
- 3
- 4
- 5
- 6
- Other

**3**. How would you describe your gender?

- Man
- Non-binary
- Woman
- Prefer not to say
- In another way (specify, if you wish)

**4.** What is your age range?

- 16-18
- 19-25
- 26-35
- 36-45
- 46-55
- >55

**5.** Do you consider yourself to have a disability?

- Yes
- No
- Prefer not to say

**6.** What is your ethnic group? (Please choose one option that best describes your ethnic group or background?

- Unsure what options are correct to use here

1. To what extent do you agree or disagree with the following statements about the teaching culture?

|  | Definitely disagree | Mostly disagree | Neither agree nor disagree | Mostly agree | Definitely agree | Not applicable |
| --- | --- | --- | --- | --- | --- | --- |
| RCSI is focused on teaching and learning strategies |  |  |  |  |  |  |
| I feel that my needs in relation to teaching and learning are addressed |  |  |  |  |  |  |
| I am aware of opportunities to become involved in peer to peer learning |  |  |  |  |  |  |

1. If you have any additional comments about the teaching culture please add them in here:
2. What, if anything, has been the one most positive aspect of your programme so far?
3. What, if anything, is the one top area in which your experience of your programme so far could be improved?
4. Thinking about the Covid-19 pandemic and lockdown, to what extent do you agree or disagree that:

|  | Definitely disagree | Mostly disagree | Neither agree nor disagree | Mostly agree | Definitely agree | Not applicable |
| --- | --- | --- | --- | --- | --- | --- |
| Communications from my institution in relation to the Covid-19 pandemic were appropriate and clear |  |  |  |  |  |  |
| I have received the support I need from my institution in relation to the Covid-19 pandemic |  |  |  |  |  |  |
| My institution has worked to ensure the quality of my academic experience during the Covid-19 pandemic |  |  |  |  |  |  |

1. If you have any additional comment about your institution’s response to the Covid-19 pandemic, please write them here:
2. Of the following teaching methods, please select all that were offered to you during the Covid-19 pandemic:

- In person teaching
- Online lectures
- Online tutorials/small group teaching
- Pre-recorded lectures
- Other (please specify):

1. Which of the following best describes your access to a device in order to access online learning:

- I do not have access to a pc/laptop/device currently
- I have access to my own pc/laptop/device – nobody else uses it
- I have access to a shared pc/laptop/device which is also used by other family members or housemates
- Other (please specify)

1. When learning from home, please rate the set-up of where you typically engage with your online learning or other coursework, with regards to the following areas:

|  | Very Poor | Below Average | Average | Above Average | Excellent |
| --- | --- | --- | --- | --- | --- |
| Privacy (e.g. in a separate room to other members of the household) |  |  |  |  |  |
| Desk and comfortable chair |  |  |  |  |  |
| Good lighting |  |  |  |  |  |
| Quiet workspace |  |  |  |  |  |
| Wi-Fi access |  |  |  |  |  |

1. Any further comments on your learning environment at home?
2. Please read the following statements and select the most appropriate response based on your experience of learning during Covid-19:

|  | Strongly disagree | Disagree | Neutral | Agree | Strongly agree |
| --- | --- | --- | --- | --- | --- |
| I felt motivated when learning from home |  |  |  |  |  |
| I was able to structure my day well when learning from home |  |  |  |  |  |
| I found it easy to access the online classes and learning materials |  |  |  |  |  |
| I found the transition to learning from home easy to manage |  |  |  |  |  |
| I felt comfortable reaching out to my tutors and lecturers if I had a question about my online learning |  |  |  |  |  |
| I felt comfortable engaging during my online classes (e.g. verbally or using the message/chat function) |  |  |  |  |  |

1. Please select any of the following techniques that you think would improve a student’s experience of online learning. You may select as many as you like.

- The use of breakout rooms in lectures where students work in smaller groups and then return to the lecture to discuss their work
- The use of ungraded quizzes during lectures to increase engagement and identify any material that needs more focus
- Cluster lectures earlier in the day when there is less “zoom fatigue” and schedule interactive sessions for later in the day
- Flipped classroom method: students are given material to look at before class and then work through it during a live online session
- Distribute a case before the online lecture or tutorial and incorporate it into the online class

1. Please add any suggestions you may have to make online learning more interactive and engaging:
2. In your experience, what is the most suitable length for an online lecture?
3. Have you used other tools or technologies to assist you with your learning during Covid-19? If yes, please give examples.
4. What, if any, were the top 3 challenges you faced when learning during Covid-19?
5. What, if anything, did you like about learning during Covid-19?
6. What, if anything, do you think could be improved with regards to the teaching you received during Covid-19?
7. Please indicate the average amount of time spent on social media each day during the time periods indicated below:

|  | 0-1 hour/day | 1-3 hours/day | 3-5 hours/day | >5 hours/day |
| --- | --- | --- | --- | --- |
| Pre-COVID-19 |  |  |  |  |
| During COVID-19 |  |  |  |  |

1. Has your usage of social media during COVID-19 affected your study schedule?
   - - Yes
     - No
     - Not applicable/I’m not on social media
2. If yes, please explain:
3. How frequently do you engage in physical activity during an average week e.g. going for a walk or run, workout session, weight training, team sports etc.?

|  | Rarely | 1-2 times/week | 3-4 times/week | >4 times/week |
| --- | --- | --- | --- | --- |
| Pre COVID-19 |  |  |  |  |
| During COVID-19 |  |  |  |  |

1. How many hours of sleep on average would you get each night?

|  | <5 hours/night | 5-7 hours/night | 7-9 hours/night | >9 hours/night |
| --- | --- | --- | --- | --- |
| Pre COVID-19 |  |  |  |  |
| During COVID-19 |  |  |  |  |

1. How would you rate the quality of your sleep on an average night?

|  | Very poor | Poor | Good | Very good |
| --- | --- | --- | --- | --- |
| Pre COVID-19 |  |  |  |  |
| During COVID-19 |  |  |  |  |

1. Has COVID-19 lead to changes in your lifestyle (e.g. sleep, exercise, hobbies, socialising)?

- Yes
- No
- I don’t know

1. If yes, has this impacted your ability to study effectively? Please explain your answer.
2. If you have any further comments or feedback on your experiences of attending university during COVID-19 then please write them here.

Thank you for taking the time to complete this survey.
