# Supplementary material for: Perspectives of educators and students on the efficacy of online teaching and learning strategies employed during COVID-19 in a health sciences institution
Source: Ir J Med Sci. 2024 Aug 16;193(6):3045–51. doi: 10.1007/s11845-024-03773-8 (PMC11666614; doi:10.1007/s11845-024-03773-8)
Supplement: Supplementary file 2 — Supplementary file2 (DOCX 38.0 KB) [file 11845_2024_3773_MOESM2_ESM.docx]

Many thanks for agreeing to participate in the study entitled **“The impact of COVID-19 on the teaching and learning strategies in a Health Sciences institution”.**

Throughout the last year, RCSI educators have reshaped how they interact with their students and have quickly adapted to a new way of teaching. The Covid-19 pandemic has forced educators everywhere to deliver lessons remotely, or in line with public health requirements. Despite these challenges, RCSI teaching staff have made enormous efforts to ensure students continue to receive a superb education. A year on, we would like to hear from tutors about their experiences during this time. Our hope is that this information can be used to ensure university students continue to receive an excellent education as we continue to live alongside Covid-19. Additionally, it will allow us to put in place the support and resources that would benefit teaching staff at this time.

**Principal investigator**: Gozie Offiah, Senior Lecturer, Department of Surgery, RCSI

**Co-investigators**: Juliette Duff, Tutor, Department of Surgery, RCSI

If you require a copy of the Participant Information Leaflet, please email gozieoffiah@rcsi.ie

The survey itself will follow after the consent, and should take 10-15 minutes to complete.

**Consent Form**

Having read the information leaflet sent to you by email, please confirm your consent by selecting Yes or No to the following questions:

- I have read and understood the Information provided about this research project.

Yes No

- I understand that my participation is voluntary.

Yes No

- I understand that I don’t have to take part in this study and that I can opt out at any time. I understand that I don’t have to give a reason for opting out and I understand that opting out won’t affect my education in RCSI.

Yes No

- I understand that I will not be paid for taking part in this study.

Yes No

- I am aware of the potential risks and benefits of this research study.

Yes No

- I consent to take part in this research study having been fully informed of the risks and benefits.

Yes No

- I give informed consent to have my data processed as part of this research study.

Yes No

With respect to possible future research related to the current study, please answer A or B here below:

1. I do not consent for this data to be used for other research studies in the future
2. I give permission for data to be stored for possible future research without further consent being required but only if the research is approved by a Research Ethics Committee

**Demographics:**

**1.** What course(s) do you teach? (e.g. Physiotherapy, Undergraduate Medicine etc.)

**2.** What year(s) do you teach? (Select all that apply)

- 1
- 2
- 3
- 4
- 5
- 6
- Other

**3.** What is your gender?

- Man
- Woman
- Prefer not to say

1. Of the following teaching methods, please select the one which best describes your role as an educator during the Covid-19 pandemic:

- In person teaching only
- Online teaching only
- Both in-person and online teaching
- Other (please specify):

1. Have you noticed a difference in how engaged students are when learning online versus in person?

- Yes, students are more engaged online
- Yes, students are less engaged online
- I have not noticed a difference
- Not applicable

1. Were there any strategies that you used to keep students engaged when teaching remotely? Please include them here.
2. Did you use breakout rooms during online classes?

- Yes
- No
- Not applicable

1. If you answered yes to Q. 4, please record your level of agreement with this statement: “*Breakout rooms improved the level of student interaction with the online class.”*

- Strongly disagree
- Disagree
- Neutral
- Agree
- Strongly agree

1. If you answered yes to Q. 4, please record your level of agreement with this statement: “*Breakout rooms helped me to identify parts of the syllabus that students found difficult.”*

- Strongly disagree
- Disagree
- Neutral
- Agree
- Strongly agree

1. If you answered yes to Q. 4, what worked well when using breakout rooms?
2. If you answered yes to Q. 4, what did not work well when using breakout rooms?
3. If you answered no to Q. 4, was there any particular reason why you did not use breakout rooms?
4. Did you use ungraded online quizzes during online classes?

- Yes
- No
- Not applicable

1. If you answered yes to Q. 10, please record your level of agreement with this statement: “*Ungraded online quizzes improved the level of student interaction with the online class.”*

- Strongly disagree
- Disagree
- Neutral
- Agree
- Strongly agree

1. If you answered yes to Q. 10, please record your level of agreement with this statement: “*Ungraded online quizzes helped me to identify parts of the syllabus that students found difficult.”*

- Strongly disagree
- Disagree
- Neutral
- Agree
- Strongly agree

1. If you answered yes to Q. 10, what worked well when using ungraded online quizzes?
2. If you answered yes to Q. 10, what did not work well when using ungraded online quizzes?
3. If you answered no to Q. 10, was there any particular reason why you did not use ungraded online quizzes?
4. Did you use the flipped classroom method, where students are given material before class and then work on it during a live class?

- Yes
- No
- Not applicable

1. If you answered yes to Q. 16, please record your level of agreement with this statement: “*The flipped classroom method improved the level of student interaction with the online class.”*

- Strongly disagree
- Disagree
- Neutral
- Agree
- Strongly agree

1. If you answered yes to Q. 16, please record your level of agreement with this statement: “*The flipped classroom method helped me to identify parts of the syllabus that students found difficult.”*

- Strongly disagree
- Disagree
- Neutral
- Agree
- Strongly agree

1. If you answered yes to Q. 16, what worked well when using the flipped classroom method?
2. If you answered yes to Q. 16, what did not work well when using the flipped classroom method?
3. If you answered no to Q. 16, was there any particular reason why you did not use the flipped classroom method?
4. If applicable, please list any other teaching strategies you used when teaching remotely and comment on their advantages and/or disadvantages.
5. Did you provide students with recordings of lectures that were given live?

- Yes
- No
- Not applicable

1. Were there any advantages or disadvantages to providing students with recordings of live lectures?
2. Did you find it harder to build rapport with students when teaching online compared with teaching in person?

- Yes, it was harder than when teaching in person
- No, it was easier than when teaching in person
- I did not notice a difference

1. Did you use any techniques to help build a rapport with the students when teaching online?
2. What, if anything, were benefits of teaching students remotely?
3. What, if anything, were the challenges of teaching students remotely?
4. How frequently did you deliver the following in-person teaching during COVID-19?

|  | Not applicable | Applicable but never | <1 time/month | 1-3 times/month | 1-3 times/week | >3 times/week |
| --- | --- | --- | --- | --- | --- | --- |
| Face-to-face lectures |  |  |  |  |  |  |
| Small group tutorials |  |  |  |  |  |  |
| Bedside tutorials |  |  |  |  |  |  |
| SIM learning |  |  |  |  |  |  |
| Clinical or practical skills |  |  |  |  |  |  |
| Teaching while attached to your team/ward |  |  |  |  |  |  |

1. Were there any other types of in-person teaching you delivered during COVID-19? (please include frequency of this teaching)
2. Please choose your response to this statement about in-person teaching during COVID-19: *“I was able to deliver in-person teaching effectively while following the public health guidelines”* (public health guidelines in this context mean 2m social distancing, with masks and appropriate hand hygiene):

- Strongly disagree
- Disagree
- Neutral
- Agree
- Strongly agree
- (not applicable)

1. Did you have to adapt your in-person teaching strategies due to COVID-19/public health guidelines?

- Yes
- No
- Neutral
- Not applicable

1. If yes, what adaptations did you make?
2. When teaching students in-person during COVID-19 (in accordance with public health guidelines), how would you describe students’ engagement levels compared to before the pandemic?

- Students were equally engaged with in-person teaching during COVID-19 compared to before the pandemic
- Students were less engaged with in-person teaching during COVID-19 compared to before the pandemic
- Students were more engaged with in-person teaching during COVID-19 compared to before the pandemic
- Not applicable

1. Please comment on how frequently you received feedback this year on your role as an educator:

|  | Never | <1 time/month | 1-3 times/month | 1-3 times/week | >3 times/week |
| --- | --- | --- | --- | --- | --- |
| Informal feedback from clinical or university supervisor |  |  |  |  |  |
| Informal feedback from student(s) |  |  |  |  |  |
| Formal feedback from clinical or university supervisor |  |  |  |  |  |
| Formal feedback from student(s) |  |  |  |  |  |

1. Has your teaching style changed in response to the pandemic?

- Yes
- No
- Not applicable

1. If yes, how has it changed?
2. Do you think a train the trainer course at the start of the year with a focus on teaching strategies would be helpful?

- Yes
- No
- Neutral

1. If you have any further comments on your experiences as an educator during Covid-19 then please add them here.

Thank you for taking the time to complete this survey.
